# Correlation analysis between gut microbiota characteristics and melasma

**DOI:** 10.3389/fmicb.2022.1051653

**Published:** 2022-11-17

**Authors:** Cong Liu, Dan He, Anye Yu, Yaru Deng, Li Wang, Zhiqi Song

**Affiliations:** ^1^Department of Dermatology, First Affiliated Hospital of Dalian Medical University, Dalian, China; ^2^Department of Pathogenobiology, Jilin University Mycology Research Center, Key Laboratory of Zoonosis Research, Ministry of Education, College of Basic Medical Sciences, Jilin University, Changchun, China

**Keywords:** melasma, gut microbiota, *Collinsella* spp., estrogen metabolism, β-glucuronidase

## Abstract

In recent years, many studies have shown that the gut microbiota can affect the occurrence and development of a variety of human diseases. A variety of skin diseases are related to the regulation of the gut–skin axis, such as psoriasis, atopic dermatitis, and acne. Gut microbial dysbiosis can promote the development of these diseases. The gut microbiota can affect estrogen metabolism, β-glucuronidase secreted by the gut microbiota can promote the reabsorption of estrogen by the gut, and estrogen is transported to other parts of the body through the circulatory system. The occurrence and development of melasma are closely related to abnormal metabolism of estrogen. The relationship between the structure of the gut microbiota and melasma remains unclear. Epidemiological surveys were conducted in patients with melasma and healthy subjects (control group) in this study. The feces were collected for 16S rRNA sequencing analysis of the gut microbiota. To compare the similarities and differences in species diversity of the gut microbiota between these two groups, we calculated the α-diversity and β-diversity indices and analyzed the differences between them. We found that the abundance of *Collinsella* spp., *Actinomyces* spp. (belonging to Actinobacteria), *Parabacteroides* spp., *Bacteroides* spp., *Paraprevotella* spp. (belonging to Bacteroidetes), *Blautia* spp., and *Roseburia* spp. (belonging to Firmicutes) in the melasma group were significantly different compared with that in the healthy group. The largest difference was found in Actinobacteria (*p* < 0.05), and there were also significant differences in the abundance of Coriobacteriia, Actinobacteria, Coriobacteriales, Coriobacteriaceae, and *Collinsella* spp. between the two groups (all *p* < 0.05). Many of these differences in the microbiota were closely related to the production of β-glucuronidase and the regulation of estrogen synthesis or metabolism. Changes in the gut microbiota structure and the biological effects of *Collinsella* spp. in the microbiota in patients with melasma can play an important role in the occurrence and development of melasma by affecting the body’s estrogen metabolism. This study provides a theoretical basis and experimental data reference for future studies on the relationship between the gut microbiota and melasma, and may be helpful for the prevention and treatment of melasma.

## Introduction

The gut microbiota is the largest and most complex micro-ecosystem in the human body, and it has metabolic functions that the rest of the body does not possess. In recent years, investigation of the effect of the gut microbiota on human health has received widespread attention. Numerous studies have shown that the gut microbiota is closely related to multiple systems in the human body (Adak and Khan, 2019; Qi et al., 2021). With regard to the gut microbiota and skin diseases, previous studies mainly focused on inflammatory skin diseases, such as psoriasis, atopic dermatitis, and acne (De Pessemier et al., 2021). At present, only the relationship between gut microbial dysbiosis and vitiligo has been preliminarily discussed (Ni et al., 2020). There have been few studies on non-inflammatory skin diseases, especially pigmented dermatosis.

At present, an increasing number of studies are actively examining the relationship between the gut microbiota and skin diseases, which has led to the concept of the gut–skin axis. The gut–skin axis links the gut microbiota to skin diseases through the gut barrier, inflammatory mediators, and metabolites (De Pessemier et al., 2021). How the gut microbiota affects skin diseases by regulating the gut–skin axis has become a hot topic of research. Previous studies have shown a bidirectional connection between the gut microbiota and skin homeostasis, gut microbial dysbiosis plays a special role in the pathophysiological process of the occurrence and development of a variety of inflammatory diseases. These diseases can promote the development of psoriasis, atopic dermatitis, acne, and others (Shah et al., 2013; Thrash et al., 2013; Salem et al., 2018). Additionally, the consumption of probiotics or live bacteria that benefit the gastrointestinal system may also prevent and control the occurrence of these skin diseases (Salem et al., 2018; De Pessemier et al., 2021).

Melasma is a stubborn pigmented dermatosis that is difficult to treat and easy to relapse after treatment, and it is a non-inflammatory skin disease. Melasma is more common in women than in men and can occur from puberty to menopause. The occurrence and development of melasma are closely related to estrogen concentrations (Lee, 2015; Filoni et al., 2019). Studies suggested the gut microbiota can affect estrogen metabolism (Plottel and Blaser, 2011; Flores et al., 2012a; Qi et al., 2021). The β-glucuronidase secreted by certain gut microbiota can deconjugate metabolized estrogen and phytoestrogen and promote their reabsorption by the gut. Estrogen is transported to distal parts of the body through the circulatory system, such as the skin and vagina (Baker et al., 2017). Previous studies demonstrated that the expression of estrogen receptors in skin lesions in patients with melasma is upregulated, estrogen then binds to the relevant estrogen receptors and affects the formation of melasma (Lieberman and Moy, 2008; Lee, 2015; Baker et al., 2017).

At present, the relationship between the gut microbiota and melasma is unclear. This study aimed to investigate the characteristics of the gut microbiota in patients with melasma and to examine the relationship between the gut microbiota and melasma to provide an experimental basis and theoretical support for the prevention and treatment of melasma.

## Materials and methods

### Subjects

In this study, we recruited 30 patients with melasma and 30 healthy people as controls in the First Affiliated Hospital of Dalian Medical University. All participants were from Dalian (Liaoning Province, China). We used questionnaires to collect information of the participants, such as age, sex, body mass index, marital and fertility status, menstrual status, bowel habits, sunscreen habits, dietary habits and disease conditions. The non-pregnant, non-menstruating women were retained as study participants. None of them had a history of taking special medications such as birth control pills. The clinical type of skin lesions in the patients was zygomatic. Then excluding those who had received systemic antibiotic treatment within 3 months before fecal samples were collected, and people with diseases which related to estrogen or gut microbial dysbiosis (e.g., gynecological, digestive and immune diseases). Finally, 7 patients with melasma and 10 healthy people as controls were retained. All patients with melasma were assessed by the modified Melasma Area and Severity Index (mMASI; Pandya et al., 2011) and examined by dermatologists. This study was approved by the Ethics Committee of the First Affiliated Hospital of Dalian Medical University, and all participants signed informed consent forms.

### Collection of fecal samples and DNA extraction

In all participants, fresh fecal samples were collected by fecal microbial DNA collection and preservation kits (TinyGene, Shanghai, China). All consumables were aseptic, and fecal samples were frozen and stored at −80°C after collection. Subsequently, the genomic DNA of samples was extracted by the QIAamp DNA Stool Mini Kit (Qiagen, Hilden, Germany).

### 16S rRNA amplification and sequencing

The sequences in the V4-V5 region of 16S rRNA were selected, and pair-end sequencing was performed in accordance with the requirements of Illumina Miseq high-throughput sequencing. After the target region and fusion primers were designed, two-step polymerase chain reaction (PCR) amplification was performed. The PCR product was recovered by using the AxyPrepDNA gel recovery kit (Axygen Scientific Inc., Silicon Valley, United States). Real-time fluorescence quantification was performed using an FTC-3000TM real-time PCR system (Funglyn, Shanghai, China). The PCR products from different samples were indexed and mixed at equal ratios, to complete the construction of an Miseq library. Then used for high-throughput sequencing and bioinformatics analysis.

### Microbiome analysis and statistical analysis

We distributed the sample reads from the raw data obtained by sequencing through a barcode to obtain the effective sequence of samples. After low-quality sequences at the ends were removed by Trimmomatic,^1^ Flash software^2^ was used to merge the paired reads into a sequence according to the overlap relationship between PE (Pair-end) reads. Additionally, Mothur software^3^ was used for quality control and filtering to obtain an optimized sequence. Subsequently, operational taxonomic unit (OTU) clustering was performed by UPARSE software^4^ under a similarity of 97%. Additionally, the chimera generated by PCR amplification was removed by UCHIME software, and the singleton OTUs were removed. The OTU representative sequence was compared using Mothur and the Silva 128 database, and species information was annotated. A statistical analysis of community structure was conducted at different classification levels.

We then conducted α-diversity and β-diversity analysis using Mothur. For α-diversity analysis, Chao, ACE, Shannon, and Simpson index values under different random sampling were calculated, and the non-parametric Wilcox test was used to analyze differences. For β-diversity analysis, Bray–Curtis analysis based on OTUs and species information was used, and ANOSIM was used to analyze differences. We plotted curve charts, box plots, sample clustering tree and histogram combination analysis diagram, and histograms of species distribution by using R language.

To identify differences in abundance in the gut microbiota between patients with melasma and controls, we used the t-test, the Wilcoxon non-parametric test, matastats (differentially abundant features analysis), and the linear discriminant analysis (LDA) effect size method. According to the obtained OUT or community abundance data, rigorous statistical methods were used for comparative analysis. The t-test and the Wilcoxon non-parametric test were adopted in the ggpubr package in R language. Matastats analysis used the metastas command in Mothur. In LEfSe analysis, a *p* < 0.05 (Kruskal–Wallis test) and log10[LDA] ≥2.0 were considered to indicate a significant difference in microorganisms.

## Results

### General information and epidemiological survey statistics

We collected fecal samples from patients in the melasma group (Group M, age: 39.57 ± 7.76 years) and healthy subjects in the control group (Group B, age: 36.4 ± 6.29 years). The mean mMASI score was 3.32 ± 1.15. There were significant differences in dietary habits between the two groups (*p* < 0.05). Different from the healthy people, the patients with melasma had a harmful habit of a high-fat diet. Details of the participants are shown in Table 1.

**Table 1:** Characteristics of patients with melasma and controls.

	Group M	Group B	*p* value
Age (years)	39.57 ± 7.76	36.4 ± 6.29	0.391
BMI	22.31 ± 2.69	21.57 ± 1.60	0.539
Dietary Habits*			
Greasy	3.29 ± 0.76	2.2 ± 1.03	0.024
Light	2.14 ± 0.38	3.1 ± 0.99	0.017
Yogurt/Probiotics	2.14 ± 0.69	2.9 ± 0.88	0.065
Meat	3.57 ± 0.98	2.4 ± 0.97	0.029
Vegetables	2.14 ± 0.69	3.1 ± 0.88	0.024
mMASI	3.32 ± 1.15		

* Dietary Habits: Preference of food, rated 1 to 5.

### Characteristics of the gut microbiota in patients with melasma

**Figure 1:**
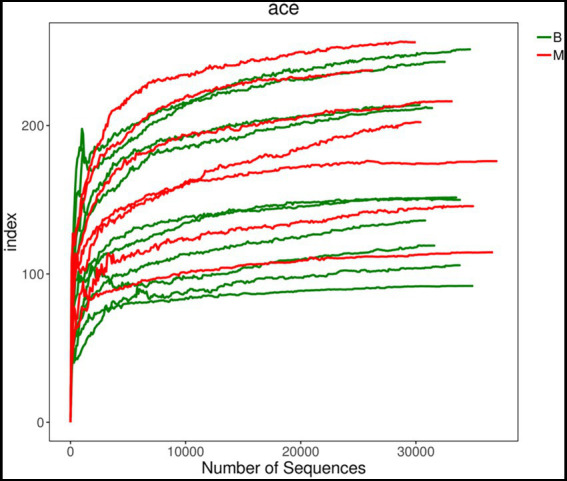
Comparison of α-diversity between Group M and Group B. ACE index dilution curve chart. There was no significant difference in α-diversity between the two groups.

**Figure 2:**
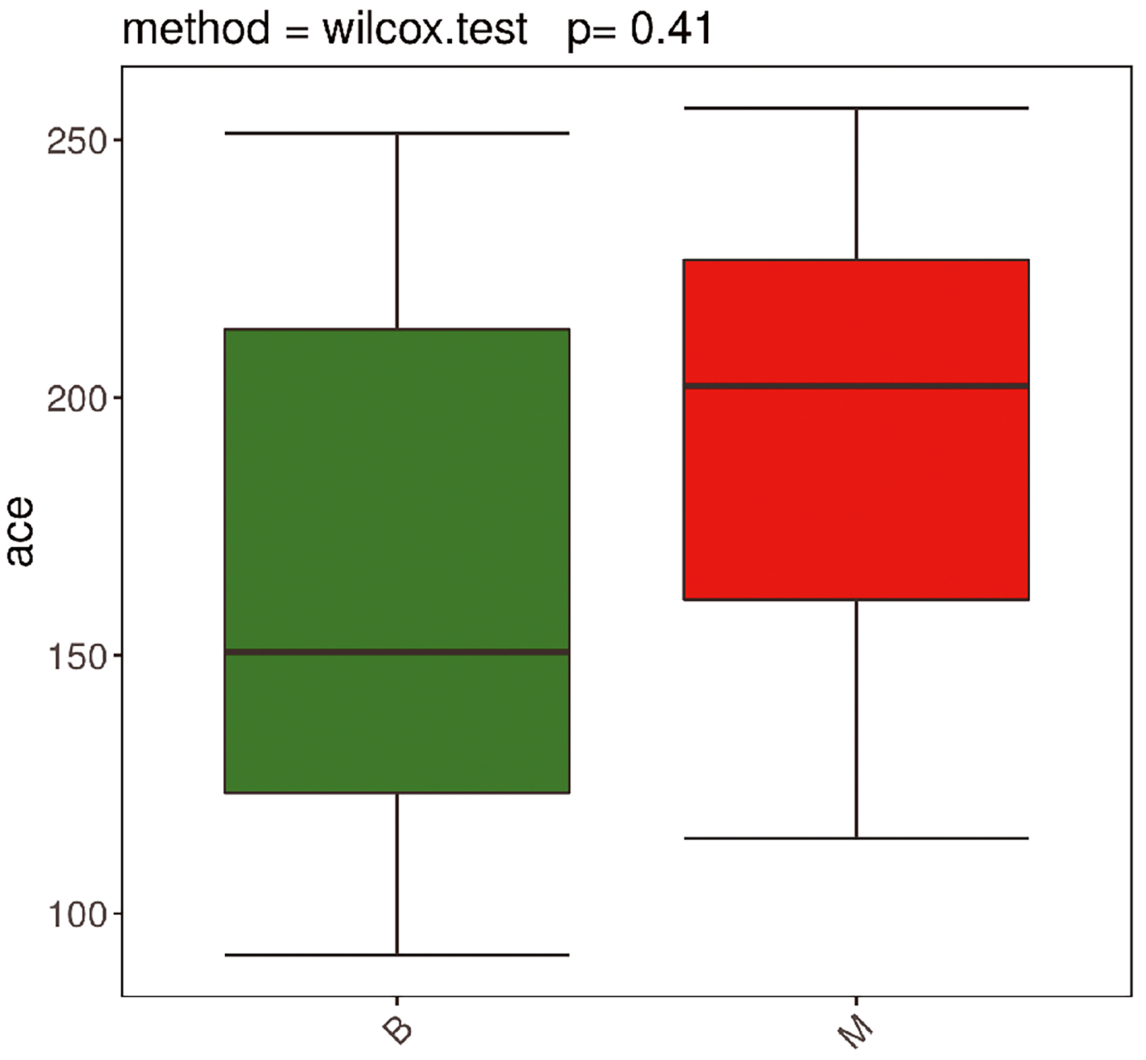
Comparison of α-diversity between Group M and Group B. ACE index non-parametric Wilcox test box plot (*p* = 0.41). There was no significant difference in α-diversity between the two groups.

To compare the similarities and differences in species diversity of the gut microbiota between the two groups, we calculated the α-diversity index and analyzed the differences between them. We found that there was no significant difference in α-diversity between the two groups (*p* = 0.41). This lack of finding suggested that there was no significant difference in species diversity of the gut bacteria between the two groups (Figures 1, 2).

**Figure 3:**
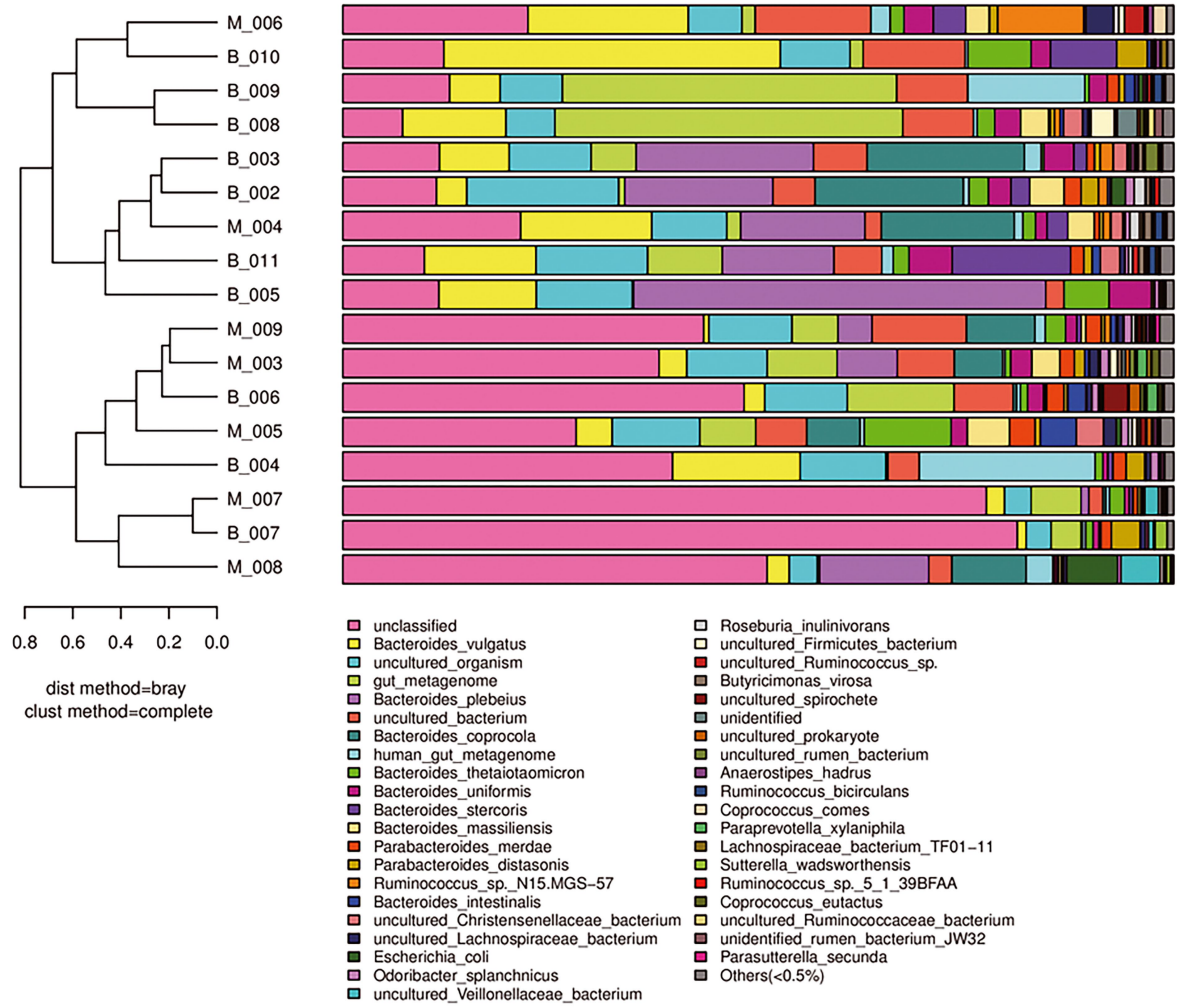
β-Diversity analysis of Group M and Group B. A sample clustering tree and histogram combination analysis diagram at the species level is shown. The left side shows a hierarchical clustering analysis between samples based on community composition. The two groups of samples show the phenomenon of convergence in the same group. The right side shows a histogram of a community structure of the corresponding samples.

To compare differences in species diversity between Group M and Group B (comparison of the similarity between samples), we conducted β-diversity analysis. A sample clustering tree and histogram combination analysis diagram (Figure 3) showed obvious convergence of samples in Group M and Group B, and samples that had similar β-diversity were clustered together. This finding suggested that there was a difference in the gut microbiota between the two groups, and the gut microbiota in Group M showed obvious similarities.

**Figure 4:**
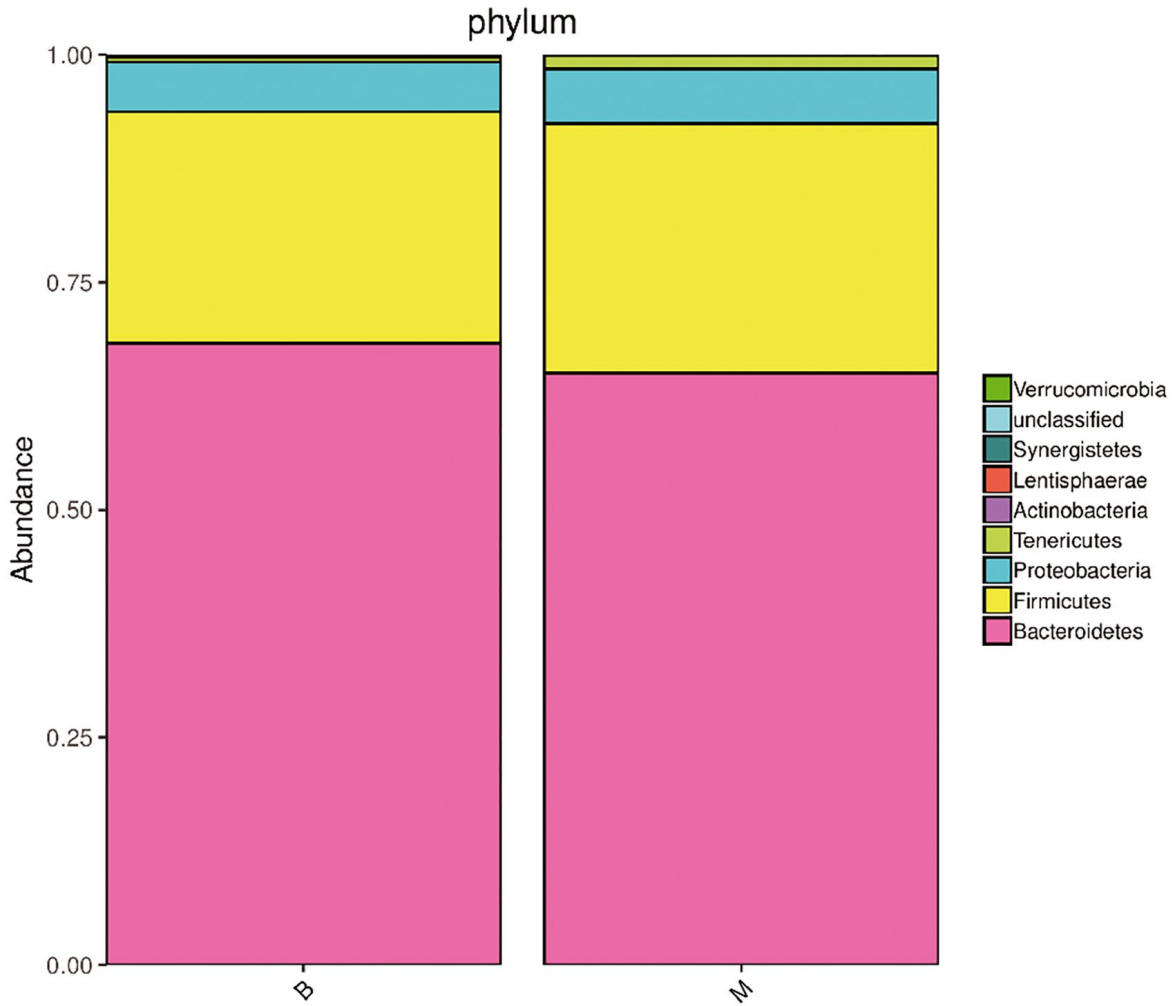
Histogram of species distribution of bacterial microbiota in Group M and Group B. Phylum level. The dominant bacteria in the gut microbiota of the two groups of samples were Bacteroidetes, Firmicutes, Proteobacteria, Tenericutes, and Actinobacteria, and their abundance was different between the two groups.

**Figure 5:**
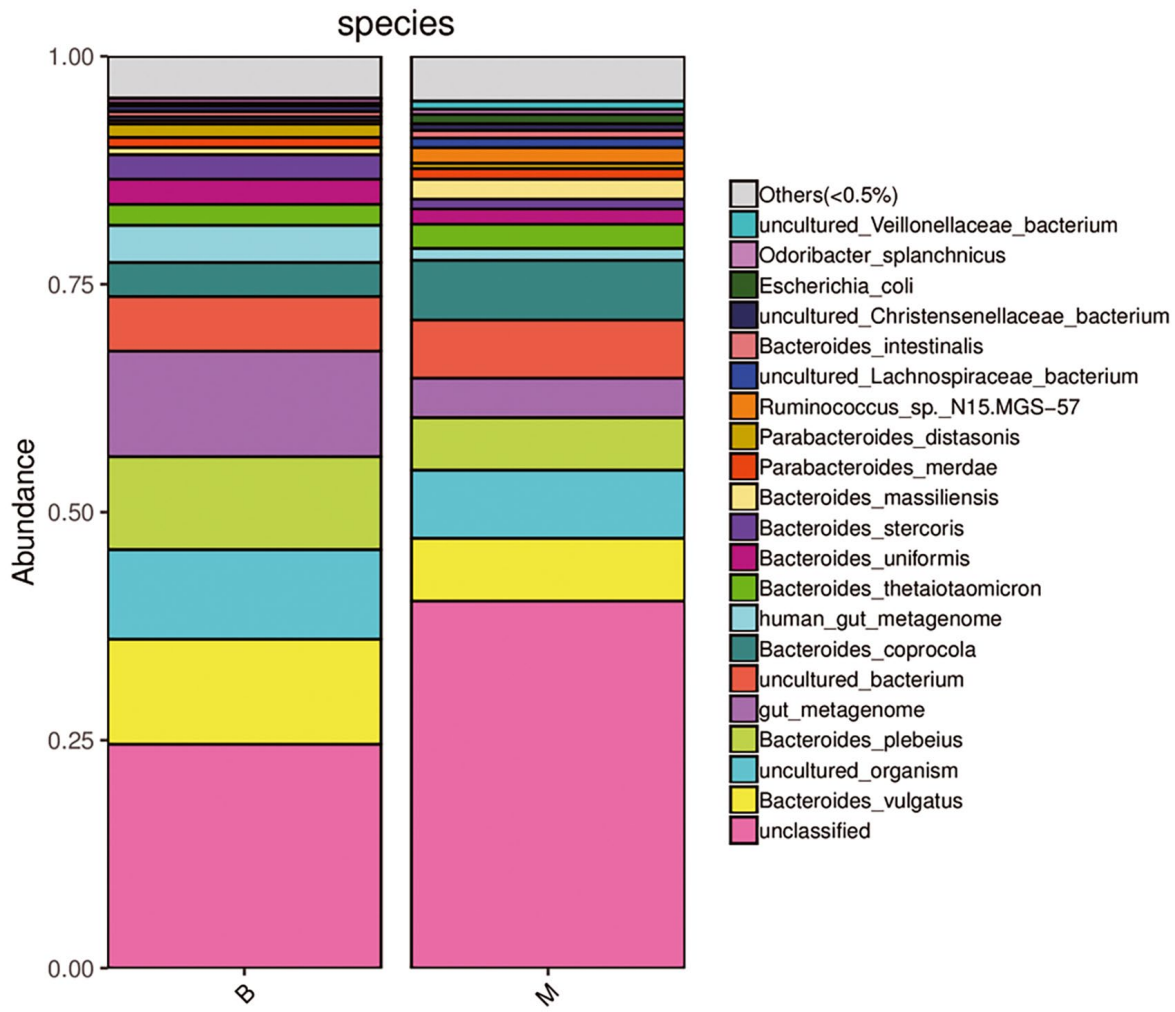
Histogram of species distribution of bacterial microbiota in Group M and Group B. Species level. Under the existing detection and identification methods, the microbial strain (pink) that could not be annotated in the gut microbiota in Group M was significantly higher than that in Group B. The overall abundance of the dominant bacterial microbiota such as *Bacteroides vulgatus* (yellow), *B. plebeius* (light green), *B. coprocola* (dark green) belonging to Bacteroidetes in Group M was also different from that in Group B.

A sample community structure histogram (Figure 3) and species distribution histogram (Figures 4, 5) show the relative abundance of different bacteria. We found that, at the phylum level, the highest abundance was Bacteroidetes, followed by Firmicutes, Proteobacteria, Tenericutes, Actinobacteria in the two groups. At the species level, the overall abundance of *Bacteroides* spp., such as *B. vulgatus*, *B. plebeius*, *B. coprocola* (belonging to Bacteroidetes) in the gut bacteria in Group M was different from that in Group B. The results of α-diversity and β-diversity analyses suggested that although the gut microbiota in Group M was not significantly different from that in Group B, the abundance of part of the microbiota was different. Additionally, these microbial strains and their biological function would be a priority for investigation.

**Figure 6:**
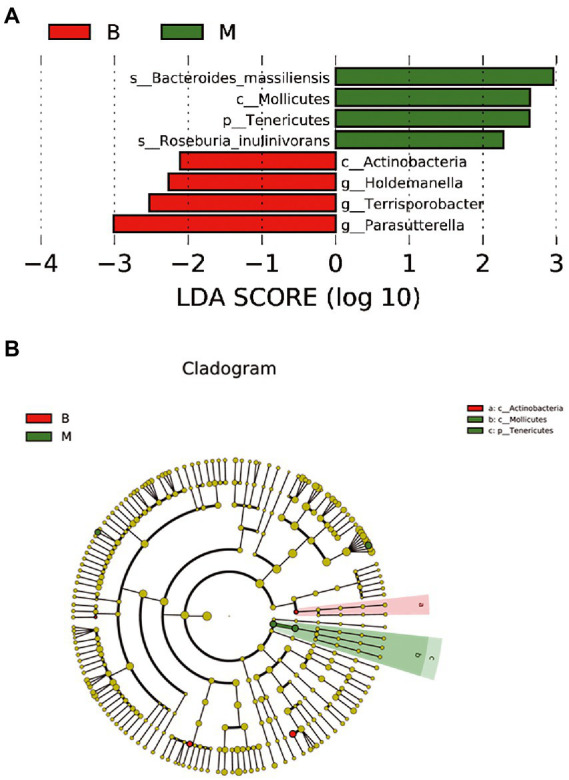
LDA effect size analysis between Group M and Group B. **(A)** Microbial species with a significant difference in abundance and a significant effect between the two groups. **(B)** A clustering tree. The concentric circles from the inside to the outside represent the classification levels of phylum, class, order, family, and genus (or species). The green and red nodes indicate microbial species that played an important role in groups M and B. The diameter of the node circle is proportional to the relative abundance of the taxonomic microbiota, which reflects a significant difference in microbial strains between the two groups and their biological correlation.

We further investigated the differences in abundance in the gut microbiota between the groups. Significant differences (*p* < 0.05) that were found under each classification level (phyla, class, order, family, genus, species, and OTU). LDA effect size analysis showed that some microbiota showed a significant difference in abundance between the two groups. The significant differences and biological relevance of microbial strains between the two groups are displayed in a clustering tree (Figure 6). Some microbial strains in the gut microbiota in Group M that showed significant differences from those in the gut microbiota in Group B are shown in Tables 2, 3.

**Table 2:** Summary of the abundance of microbial strains at different species levels between Group M and Group B.

OTU ID	Phylum	Class	Order	Family	Genus
97	Actinobacteria	Coriobacteriia	Coriobacteriales	Coriobacteriaceae	*Collinsella*
275	Actinobacteria	Coriobacteriia	Coriobacteriales	Coriobacteriaceae	
303	Actinobacteria	Actinobacteria	Actinomycetales	Actinomycetaceae	*Actinomyces*
39	Bacteroidetes	Bacteroidia	Bacteroidales	Porphyromonadaceae	*Parabacteroides*
388	Bacteroidetes	Bacteroidia	Bacteroidales	Bacteroidaceae	*Bacteroides*
11	Bacteroidetes	Bacteroidia	Bacteroidales	Bacteroidaceae	*Bacteroides*
170	Bacteroidetes	Bacteroidia	Bacteroidales	Bacteroidaceae	*Bacteroides*
120	Bacteroidetes	Bacteroidia	Bacteroidales	Prevotellaceae	*Paraprevotella*
47	Firmicutes	Clostridia	Clostridiales	Lachnospiraceae	
130	Firmicutes	Clostridia	Clostridiales	Lachnospiraceae	
179	Firmicutes	Clostridia	Clostridiales	Lachnospiraceae	*Blautia*
228	Firmicutes	Clostridia	Clostridiales	Lachnospiraceae	
79	Firmicutes	Clostridia	Clostridiales	Lachnospiraceae	
36	Firmicutes	Clostridia	Clostridiales	Lachnospiraceae	
60	Firmicutes	Clostridia	Clostridiales	Lachnospiraceae	*Roseburia*
410	Firmicutes	Clostridia	Clostridiales	Ruminococcaceae	
127	Firmicutes	Clostridia	Clostridiales	Ruminococcaceae	*Ruminiclostridium*
334	Firmicutes	Clostridia	Clostridiales	Ruminococcaceae	
381	Firmicutes	Clostridia	Clostridiales	Ruminococcaceae	
391	Firmicutes	Clostridia	Clostridiales	Ruminococcaceae	
139	Firmicutes	Clostridia	Clostridiales	Ruminococcaceae	
224	Firmicutes	Clostridia	Clostridiales	Peptostreptococcaceae	*Intestinibacter*
336	Firmicutes	Clostridia	Clostridiales	Peptostreptococcaceae	*Terrisporobacter*
403	Firmicutes	Clostridia	Clostridiales	Peptococcaceae	
370	Firmicutes	Clostridia	Clostridiales		
375	Firmicutes	Erysipelotrichia	Erysipelotrichales	Erysipelotrichaceae	
62	Firmicutes	Erysipelotrichia	Erysipelotrichales	Erysipelotrichaceae	*Holdemanella*
16	Proteobacteria	Betaproteobacteria	Burkholderiales	Alcaligenaceae	*Parasutterella*
117	Tenericutes	Mollicutes			

**Table 3:** Microbial strains with significant differences at each classification level between Group M and Group B.

	Mean relative abudance (%)		Variance		*p* value
	Group M	Group B	Group M	Group B	
Phylum					
Actinobacteria	0.0552	0.1992	0	0.000004	0.0358
Class					
Deltaproteobacteria	0.0133	0.0379	0	0	0.027611
Coriobacteriia	0.0489	0.1711	0	0.000003	0.034333
Actinobacteria	0.00630	0.02810	0.000047	0.000345	0.036
Order					
Desulfovibrionales	0.0133	0.0379	0	0	0.02696
Coriobacteriales	0.0489	0.1711	0	0.000003	0.03124
Family					
Desulfovibrionaceae	0.0133	0.0379	0	0	0.029737
Coriobacteriaceae	0.0489	0.1711	0	0.000003	0.035368
Genus					
*Parasutterella* spp.	0.4602	2.3846	0.000054	0.000596	0.015688
*Collinsella* spp.	0.0237	0.1371	0	0.000003	0.03815
*Terrisporobacter* spp.	0	0.0021	0	0	0.046446
*Holdemanella* spp.	0.11706	0.33713	0.003371	0.000353	0.012

Species with a significant difference of *p* < 0.05 were selected.

Notably, at the phylum level, the abundance of Actinobacteria in Group M was significantly lower than that in Group B (*p* < 0.05). There were also significant differences in the abundance of Coriobacteriia, Actinobacteria, Coriobacteriales, Coriobacteriaceae, and *Collinsella* spp. between the two groups (all *p* < 0.05). These findings suggested that the differential microbiota between patients with melasma and healthy subjects, and may play an important role in the occurrence of melasma. Additionally, the abundance of Bacteroidetes, Firmicutes, and Proteobacteria was different in some microbial strains between the two groups. There were also significant differences in the abundance of Tenericutes, Mollicutes, Deltaproteobacteria, Desulfovibrionales, Desulfovibrionaceae, *Terrisporobacter* spp., *Holdemanella* spp., *Parasutterella* spp. between the two groups. This finding suggested that these genera may play a co-regulatory role in the occurrence and development of melasma (Table 3; Figure 6).

## Discussion

The gut microbiota, which has recently received a lot of attention, is related to inflammatory skin diseases (De Pessemier et al., 2021). However, there have been limited studies on the gut microbiota and pigmented dermatosis (Ni et al., 2020), and no studies on melasma have been reported.

The gut microbiota secretes β-glucuronidase to dissociate estrogen in the early stage, promotes its reabsorption of estrogen into the blood through the intestines, and transports estrogen to distal parts of the body to bind with the estrogen receptor to take effect. Therefore, the gut microbiota affects the metabolism of human estrogen. In this process, the gut microbiota and estrogen act on the distal effector sites through circulatory metabolism (Baker et al., 2017; De Pessemier et al., 2021). Studies have shown that estrogen concentrations in patients with melasma are abnormal compared with those in the healthy population (Pérez et al., 1983; Lee, 2015). Additionally, the expression of estrogen receptors in skin lesions in patients with melasma is upregulated (Lieberman and Moy, 2008; Lee, 2015), which further confirms the role of estrogen in this disease. Whether the gut microbiota in patients with melasma is different from that in the healthy population, whether there are some characteristic components of the microbiota, and whether these will affect the occurrence and development of melasma, are important issues that need to be examined.

In the healthy human intestines, the relative abundance of Bacteroidetes and Firmicutes accounts for more than 90% of the gut microbiota, and they play a major role in maintaining gut homeostasis, while Proteobacteria and Actinobacteria only account for nearly 10% (Arumugam et al., 2011; Segata et al., 2012). Bacteroidetes, Firmicutes, Proteobacteria, and Actinobacteria constitute the four main phyla in the human gut microbiota (Arumugam et al., 2011; Segata et al., 2012). The abundance of Bacteroidetes, Firmicutes, Proteobacteria, and Actinobacteria in the intestines in patients with melasma was different from that in the healthy population, especially for Actinobacteria. Many of the differential microbiota are related to the metabolic regulation of estrogen in the body, indicating that they may play an important role in the occurrence of melasma.

Although Actinobacteria accounts for a small proportion of the gut microbiota, it showed the most significant difference in abundance between patients with melasma and healthy people. Actinobacteria also plays an important role in maintaining homeostasis of the gut environment (Binda et al., 2018). Moreover, its secondary metabolites are abundant, has important biological value, and it can produce naturally derived antibiotics, antifungals, anthelmintics, and anticancer compounds, which can be applied clinically (Barka et al., 2016). The most common Bifidobacteria in the Actinobacteria family is also a widely used probiotic (Binda et al., 2018). Our study showed that the abundance of Actinobacteria in patients with melasma was significantly lower than that in healthy people. There were also significant differences in the Coriobacteriia, Actinobacteria, Coriobacteriales, Coriobacteriaceae, and *Collinsella* spp. between the two groups. Actinobacteria can participate in the aerobic degradation of estrogen (Yu et al., 2013; Wu et al., 2019). Coriobacteriaceae has also been reported to be involved in the synthesis of the phytoestrogen S-equol. S-equol reduces the incidence of menopausal symptoms, osteoporosis, skin aging, hair loss, prostate cancer, and ovarian cancer by selectively activating estrogen receptors, and has a variety of biological and clinical uses (Lee et al., 2018).The findings suggest that the differential microbiota may play an important role in the occurrence of melasma as a characteristic part of the microbiota in these patients. A decrease in the abundance of Actinobacteria in the microbiota may affect estrogen concentrations of the body, and then affect the occurrence and development of melasma. This possibility could provide a new target for the prevention and treatment of melasma.

As dominant members of the microbiota with the highest proportion in the human gut, Bacteroidetes and Firmicutes are important for biological function. Studies have shown that Bacteroidetes degrades polysaccharides, and may promote inflammation and stimulate angiogenesis (Johnson et al., 2017). Firmicutes member Lactobacillus is also a common probiotic that helps the body absorb energy and fat (Jeong et al., 2017). Disorder of Bacteroidetes and Firmicutes are also been related to skin diseases such as psoriasis (Sikora et al., 2020). There have been reports that Clostridia (belonging to Firmicutes) affects estrogen concentrations in the body through β-glucuronidase. Moreover, estrogen can reverse changes in the microbiota, forming a two-way regulation (Flores et al., 2012a,b). In our study, we found that the abundance of Bacteroidetes and Firmicutes and *Bacteroides* spp. (belonging to Bacteroidetes), Clostridia and *Blautia* spp. (belonging to Firmicutes) were different between patients with melasma and healthy people. They can regulate the secretion and activity of β-glucuronidase, thereby affecting the body’s estrogen concentrations (Gloux et al., 2011; Flores et al., 2012a; Pellock et al., 2018; Creekmore et al., 2019; Dai et al., 2019; Ibrahim et al., 2019; Zhang et al., 2019). These results indicate that the differential microbiota may play a co-regulatory role in the occurrence and development of melasma.

In addition, we conducted an epidemiological study on patients with melasma, and found that these patients, unlike healthy people, had a bad habit of a high-fat diet. Studies have shown that different dietary patterns can affect the growth and reproduction of Actinobacteria, Bacteroidetes and Firmicutes (Brahe et al., 2015; Guo et al., 2017). In this study, we found gut microbial dysbiosis in patients with melasma, with a decrease in Actinobacteria and Bacteroidetes and an increase in Firmicutes, which may have been related to the patients’ high-fat diet. This finding suggested that patients should improve their dietary habits or consume probiotics (e.g., Bifidobacterium, Lactobacillus) to regulate the gut microbiota (Gibson and Roberfroid, 1995; Menon et al., 2013). Adjustment of the ratios of Actinobacteria, Bacteroidetes, and Firmicutes in the human gut and improving the structure of the gut microbiota may be an auxiliary means to improve the patient’s condition or prevent the occurrence of diseases.

In conclusion, the gut microbiota structure in patients with melasma is different to that of healthy people. The abundance of *Collinsella* spp., *Actinomyces* spp. (belonging to Actinobacteria), *Parabacteroides* spp., *Bacteroides* spp., *Paraprevotella* spp. (belonging to Bacteroidetes), *Blautia* spp., *Roseburia* spp. (belonging to Firmicutes), and that of other members of the microbiota are different from those of healthy people. In particular, *Collinsella* spp. is a characteristic member of the microbiota in patients with melasma. The biological effects of these differential microbiota play an important role in the occurrence and development of melasma by affecting estrogen metabolism. This study provides a theoretical basis and experimental data reference for future studies on the relationship between melasma and the gut microbiota. Add the gut–skin axis in the understanding of melasma pathogenesis, may be helpful for the prevention and treatment of melasma.

## Data availability statement

The data presented in the study are deposited in the NCBI’s Gene Expression Omnibus repository, accession number GSE214430.

## Ethics statement

The studies involving human participants were reviewed and approved by the Ethics Committee of the First Affiliated Hospital of Dalian Medical University. The patients/participants provided their written informed consent to participate in this study.

## Author contributions

LW and ZS: conceptualization, design, and review and editing. CL, AY, and YD: collect the fecal samples and epidemiological survey. DH: data analysis. CL and DH: original manuscript. All authors contributed to the article and approved the submitted version.

## Funding

This study was supported by grants from the National Natural Science Foundation of China (82073416).

## Conflict of interest

The authors declare that the research was conducted in the absence of any commercial or financial relationships that could be construed as a potential conflict of interest.

## Publisher’s note

All claims expressed in this article are solely those of the authors and do not necessarily represent those of their affiliated organizations, or those of the publisher, the editors and the reviewers. Any product that may be evaluated in this article, or claim that may be made by its manufacturer, is not guaranteed or endorsed by the publisher.

## Footnotes

^1^ http://www.usadellab.org/cms/index.php?page=trimmomatic

^2^ http://ccb.jhu.edu/software/FLASH/index.shtml

^3^ http://mothur.org/

^4^ https://drive5.com/usearch/manual8.1/uparse_pipeline.html

## References

[ref1] Adak A. Khan M. R. (2019). An insight into gut microbiota and its functionalities. Cell. Mol. Life Sci. 76, 473–493. doi: 10.1007/s00018-018-2943-4, PMID: 30317530 PMC11105460

[ref2] Arumugam M. Raes J. Pelletier E. Le Paslier D. Yamada T. Mende D. R.. (2011). Enterotypes of the human gut microbiome. Nature 473, 174–180. doi: 10.1038/nature09944, PMID: 21508958 PMC3728647

[ref3] Baker J. M. Al-Nakkash L. Herbst-Kralovetz M. M. (2017). Estrogen-gut microbiome axis: physiological and clinical implications. Maturitas 103, 45–53. doi: 10.1016/j.maturitas.2017.06.025, PMID: 28778332

[ref4] Barka E. A. Vatsa P. Sanchez L. Gaveau-Vaillant N. Jacquard C. Meier-Kolthoff J. P.. (2016). Taxonomy, physiology, and natural products of Actinobacteria. Microbiol. Mol. Biol. Rev. 80, 1–43. doi: 10.1128/MMBR.00019-15, PMID: 26609051 PMC4711186

[ref5] Binda C. Lopetuso L. R. Rizzatti G. Gibiino G. Cennamo V. Gasbarrini A. (2018). Actinobacteria: a relevant minority for the maintenance of gut homeostasis. Dig. Liver Dis. 50, 421–428. doi: 10.1016/j.dld.2018.02.012, PMID: 29567414

[ref6] Brahe L. K. Le Chatelier E. Prifti E. Pons N. Kennedy S. Blædel T.. (2015). Dietary modulation of the gut microbiota--a randomised controlled trial in obese postmenopausal women. Br. J. Nutr. 114, 406–417. doi: 10.1017/S0007114515001786, PMID: 26134388 PMC4531470

[ref7] Creekmore B. C. Gray J. H. Walton W. G. Biernat K. A. Little M. S. Xu Y.. (2019). Mouse gut microbiome-encoded β-Glucuronidases identified using metagenome analysis guided by protein. Structure 4, e00452–e00419. doi: 10.1128/mSystems.00452-19, PMID: 31455640 PMC6712278

[ref8] Dai S. Pan M. El-Nezami H. S. Wan J. Wang M. F. Habimana O.. (2019). Effects of lactic acid bacteria-fermented soymilk on Isoflavone metabolites and short-chain fatty acids excretion and their modulating effects on gut microbiota. J. Food Sci. 84, 1854–1863. doi: 10.1111/1750-3841.14661, PMID: 31206699

[ref9] De Pessemier B. Grine L. Debaere M. Maes A. Paetzold B. Callewaert C. (2021). Gut-skin axis: current knowledge of the interrelationship between microbial dysbiosis and skin conditions. Microorganisms 9, 9:353. doi: 10.3390/microorganisms9020353, PMID: 33670115 PMC7916842

[ref10] Filoni A. Mariano M. Cameli N. (2019). Melasma: how hormones can modulate skin pigmentation. J. Cosmet. Dermatol. 18, 458–463. doi: 10.1111/jocd.12877, PMID: 30779300

[ref11] Flores R. Shi J. Fuhrman B. Xu X. Veenstra T. D. Gail M. H.. (2012a). Fecal microbial determinants of fecal and systemic estrogens and estrogen metabolites: a cross-sectional study. J. Transl. Med. 10:253. doi: 10.1186/1479-5876-10-253, PMID: 23259758 PMC3552825

[ref12] Flores R. Shi J. Gail M. H. Gajer P. Ravel J. Goedert J. J. (2012b). Association of fecal microbial diversity and taxonomy with selected enzymatic functions. PLoS One 7:e39745. doi: 10.1371/journal.pone.0039745, PMID: 22761886 PMC3386201

[ref13] Gibson G. R. Roberfroid M. B. (1995). Dietary modulation of the human colonic microbiota: introducing the concept of prebiotics. J. Nutr. 125, 1401–1412. doi: 10.1093/jn/125.6.1401, PMID: 7782892

[ref14] Gloux K. Berteau O. El Oumami H. Béguet F. Leclerc M. Doré J. (2011). A metagenomic β-glucuronidase uncovers a core adaptive function of the human intestinal microbiome. Proc. Natl. Acad. Sci. U. S. A. 108, 4539–4546. doi: 10.1073/pnas.1000066107, PMID: 20615998 PMC3063586

[ref15] Guo X. Li J. Tang R. Zhang G. Zeng H. Wood R. J.. (2017). High fat diet alters gut microbiota and the expression of Paneth cell-antimicrobial peptides preceding changes of circulating inflammatory cytokines. Mediat. Inflamm. 2017, 9474896–9474899. doi: 10.1155/2017/9474896, PMID: 28316379 PMC5339499

[ref16] Ibrahim A. Hugerth L. W. Hases L. Saxena A. Seifert M. Thomas Q.. (2019). Colitis-induced colorectal cancer and intestinal epithelial estrogen receptor beta impact gut microbiota diversity. Int. J. Cancer 144, 3086–3098. doi: 10.1002/ijc.32037, PMID: 30515752 PMC6519213

[ref17] Jeong S. Y. Kang S. Hua C. S. Ting Z. Park S. (2017). Synbiotic effects of β-glucans from cauliflower mushroom and *lactobacillus fermentum* on metabolic changes and gut microbiome in estrogen-deficient rats. Genes Nutr. 12:31. doi: 10.1186/s12263-017-0585-z, PMID: 29151980 PMC5679333

[ref18] Johnson E. L. Heaver S. L. Walters W. A. Ley R. E. (2017). Microbiome and metabolic disease: revisiting the bacterial phylum Bacteroidetes. J. Mol. Med. 95, 1–8. doi: 10.1007/s00109-016-1492-2, PMID: 27900395 PMC5187364

[ref19] Lee A. Y. (2015). Recent progress in melasma pathogenesis. Pigment Cell Melanoma Res. 28, 648–660. doi: 10.1111/pcmr.12404, PMID: 26230865

[ref20] Lee P. G. Lee S. H. Kim J. Kim E. J. Choi K. Y. Kim B. G. (2018). Polymeric solvent engineering for gram/liter scale production of a water-insoluble isoflavone derivative, (S)-equol. Appl. Microbiol. Biotechnol. 102, 6915–6921. doi: 10.1007/s00253-018-9137-8, PMID: 29948112

[ref21] Lieberman R. Moy L. (2008). Estrogen receptor expression in melasma: results from facial skin of affected patients. J. Drugs Dermatol. 7, 463–465. PMID: 18505139

[ref22] Menon R. Watson S. E. Thomas L. N. Allred C. D. Dabney A. Azcarate-Peril M. A.. (2013). Diet complexity and estrogen receptor β status affect the composition of the murine intestinal microbiota. Appl. Environ. Microbiol. 79, 5763–5773. doi: 10.1128/AEM.01182-13, PMID: 23872567 PMC3754184

[ref23] Ni Q. Ye Z. Wang Y. Chen J. Zhang W. Ma C.. (2020). Gut microbial dysbiosis and plasma metabolic profile in individuals with vitiligo. Front. Microbiol. 11:592248. doi: 10.3389/fmicb.2020.592248, PMID: 33381090 PMC7768019

[ref24] Pandya A. G. Hynan L. S. Bhore R. Riley F. C. Guevara I. L. Grimes P.. (2011). Reliability assessment and validation of the Melasma area and severity index (MASI) and a new modified MASI scoring method. J. Am. Acad. Dermatol. 64, 78–72. doi: 10.1016/j.jaad.2009.10.051, PMID: 20398960

[ref25] Pellock S. J. Walton W. G. Biernat K. A. Torres-Rivera D. Creekmore B. C. Xu Y.. (2018). Three structurally and functionally distinct β-glucuronidases from the human gut microbe Bacteroides uniformis. J. Biol. Chem. 293, 18559–18573. doi: 10.1074/jbc.RA118.005414, PMID: 30301767 PMC6290157

[ref26] Pérez M. Sánchez J. L. Aguiló F. (1983). Endocrinologic profile of patients with idiopathic melasma. J. Invest. Dermatol. 81, 543–545. doi: 10.1111/1523-1747.ep12522896, PMID: 6644096

[ref27] Plottel C. S. Blaser M. J. (2011). Microbiome and malignancy. Cell Host Microbe 10, 324–335. doi: 10.1016/j.chom.2011.10.003, PMID: 22018233 PMC3264051

[ref28] Qi X. Yun C. Pang Y. Qiao J. (2021). The impact of the gut microbiota on the reproductive and metabolic endocrine system. Gut Microbes 13, 1–21. doi: 10.1080/19490976.2021.1894070, PMID: 33722164 PMC7971312

[ref29] Salem I. Ramser A. Isham N. Ghannoum M. A. (2018). The gut microbiome as a major regulator of the gut-skin Axis. Front. Microbiol. 9:1459. doi: 10.3389/fmicb.2018.01459, PMID: 30042740 PMC6048199

[ref30] Segata N. Haake S. K. Mannon P. Lemon K. P. Waldron L. Gevers D.. (2012). Composition of the adult digestive tract bacterial microbiome based on seven mouth surfaces, tonsils, throat and stool samples. Genome Biol. 13:R42. doi: 10.1186/gb-2012-13-6-r42, PMID: 22698087 PMC3446314

[ref31] Shah K. R. Boland C. R. Patel M. Thrash B. Menter A. (2013). Cutaneous manifestations of gastrointestinal disease: part I. J. Am. Acad. Dermatol. 68, e1–e21. doi: 10.1016/j.jaad.2012.10.037 23317980

[ref32] Sikora M. Stec A. Chrabaszcz M. Knot A. Waskiel-Burnat A. Rakowska A.. (2020). Gut microbiome in psoriasis: an updated review. Pathogens 9:463. doi: 10.3390/pathogens9060463, PMID: 32545459 PMC7350295

[ref33] Thrash B. Patel M. Shah K. R. Boland C. R. Menter A. (2013). Cutaneous manifestations of gastrointestinal disease: part II. J. Am. Acad. Dermatol. 68:211.e1-33; quiz 244-246. doi: 10.1016/j.jaad.2012.10.036, PMID: 23317981

[ref34] Wu K. Lee T. H. Chen Y. L. Wang Y. S. Wang P. H. Yu C. P.. (2019). Metabolites involved in aerobic degradation of the a and B rings of estrogen. Appl. Environ. Microbiol. 85, e02223–e02218. doi: 10.1128/AEM.02223-18, PMID: 30446556 PMC6344625

[ref35] Yu C. P. Deeb R. A. Chu K. H. (2013). Microbial degradation of steroidal estrogens. Chemosphere 91, 1225–1235. doi: 10.1016/j.chemosphere.2013.01.112, PMID: 23517889

[ref36] Zhang J. Lacroix C. Wortmann E. Ruscheweyh H. J. Sunagawa S. Sturla S. J.. (2019). Gut microbial beta-glucuronidase and glycerol/diol dehydratase activity contribute to dietary heterocyclic amine biotransformation. BMC Microbiol. 19:99. doi: 10.1186/s12866-019-1483-x, PMID: 31096909 PMC6524314

